# Tranexamic Acid Is Beneficial to Patients Undergoing Open-Wedge High Tibial Osteotomy

**DOI:** 10.1155/2020/2514207

**Published:** 2020-11-04

**Authors:** De-Sheng Chen, Jia-Wang Zhu, Tong-Fu Wang, Bo Zhu, Cai-Hong Feng

**Affiliations:** Department of Tianjin Hospital, Tianjin, China

## Abstract

The purpose of this study was to investigate the efficacy of tranexamic acid (TXA) in patients undergoing open-wedge high tibial osteotomy (OWHTO). Patients from August 2018 to May 2020 were retrospectively studied. Clinical data were obtained including gender, age, height, weight, body mass index (BMI), smoking, alcohol consumption, hypertension, diabetes, history of aspirin, prepostoperative hematocrit (Hct) and hemoglobin (Hb), thrombotic events, blood transfusion requirement, hospital length of stay, size of osteotomy gap, and wound complications such as wound hematoma and infection. 52 patients were enrolled in the tranexamic acid group (TA group), and 48 patients were enrolled in the nontranexamic acid group (NTA group); there were no significant differences between both groups in terms of gender, age, BMI, preoperative Hb, size of osteotomy gap, incidence of smoking, alcohol consumption, hypertension, diabetes, history of aspirin, thrombotic events, blood transfusion requirement, and wound hematoma and infection. The mean hospital length of stay was 9.4 ± 1.0 days in the TA group and 11.0 ± 1.2 days in the NTA group (*P* < 0.001), the blood loss was 296.0 ± 128.7 ml in the TA group and 383.3 ± 181.3 ml in the NTA group (*P* < 0.05), and the postoperative Hb level was 120.8 ± 15.0 g/l in the TA group and 109.5 ± 13.8 g/l in the NTA group (*P* < 0.001). In conclusion, the administration of TXA is beneficial to patients undergoing OWHTO via decreasing hospital length of stay, reducing blood loss, and maintaining higher postoperative Hb levels.

## 1. Introduction

Knee osteoarthritis (OA) is characterized by articular cartilage lesions and underlying bone destruction [1–4]. The global prevalence of knee OA was 3.8%, and knee OA was increasingly recognised as a serious, worldwide public health concern [5–7].

Open-wedge high tibial osteotomy (OWHTO) is a reliable option for knee osteoarthritis with varus malalignment in young, active people [8–11]. This technique is easy to learn and allows precise correction during the surgical procedure; however, it creates an osteotomy gap and increases the risk of bleeding theoretically [12–14].

Perioperative blood loss could be minimized by using a tourniquet and antifibrinolytic agents such as tranexamic acid (TXA) [15–19]. TXA is a synthetic antifibrinolytic drug that inhibits the activation of plasminogen [20, 21]. Relevant studies have confirmed that TXA is effective in reducing perioperative blood loss and transfusion requirements in cardiac, obstetric, and urologic surgery [22–25]. In orthopaedic surgery, a large retrospective cohort study involving over 800000 patients undergoing total hip or knee arthroplasty revealed that the demand for allogeneic or autologous blood transfusions was decreased by up to 69% in the tranexamic acid group [26]. Our previous study revealed that alcohol consumption and BMI are important risk factors related to blood loss in OWHTO [27]; however, there is less research on the effects of TXA in patients' blood management about OWHTO. The purpose of this study was to investigate the efficacy of TXA in patients undergoing OWHTO.

## 2. Materials and Methods

### 2.1. Study Design and Patients

We conducted a retrospective cohort study of patients undergoing OWHTO at the *Tianjin Hospital* from August 2018 to May 2020. Approval from the Institutional Ethics Committee of *Tianjin Hospital* was obtained, and informed consent was obtained from each participant. The inclusion criteria for participants were as follows: (a) medial knee osteoarthritis with varus deformity requiring correction, (b) first OWHTO on the affected side, and (c) complete clinical and follow-up data. The exclusion criteria were as follows: (a) coagulation disorders, (b) contraindications to TXA, (c) recent thromboembolism or anticoagulant therapy, and (d) simultaneous bilateral HTO.

### 2.2. Perioperative Management

All patients received standard medical care, and all procedures were performed by the same surgical team. The osteotomy site was fixed with Tomofix plate system. Patients who received tranexamic acid (TA group) were administered intravenous tranexamic acid 1 g before tourniquet inflation and topical tranexamic acid 1 g at osteotomy site; patients in the nontranexamic acid group (NTA group) did not receive any additional treatment. There were no significant differences between the groups in terms of the time of tourniquet use and operative time. Postoperative antibiotic prophylaxis and analgesia were administered in all patients; low molecule weight heparin was used for the prevention of venous thromboembolism. Functional exercise was started after operation, and the blood routine examination was performed 3 days after surgery. All patients received Doppler ultrasound of both lower limbs before discharge, or anytime in suspicion of deep vein thrombosis. The decision of blood transfusion was left to the discretion of anesthesiologist and surgeon; the same discharge criteria were applied in both groups.

### 2.3. Data Collection

The demographic and clinical data of patients were collected, including gender, age, height, weight, body mass index (BMI), smoking, alcohol consumption, hypertension, diabetes, history of aspirin, the prepostoperative hematocrit (Hct) and hemoglobin (Hb), thrombotic events, blood transfusion requirement, hospital length of stay, size of osteotomy gap, and wound complications such as wound hematoma and infection.

The total blood volume was calculated using height and weight by the formula of Nadler et al. [28]. The blood loss was calculated by the Gross formula [29]. Multiple linear regression analysis was used to investigate the risk factors for blood loss in OWHTO.

### 2.4. Statistical Analysis

The measurement data were expressed as mean value ± standard deviation (SD); the prepostoperative parameters in the same group were compared using paired *t*-tests and comparisons between groups were performed using independent sample *t*-test. The count data were expressed as percentages and analyzed by Chi-square test. The SPSS 22.0 software was used for statistical analysis, and *P* < 0.05 was considered statistically significant.

## 3. Results

52 patients were enrolled in the TA group, and 48 patients were enrolled in the NTA group. All patients received optimal wound healing, and none had received blood transfusion. There were no significant differences between both groups in terms of age, BMI, preoperative Hb, and size of osteotomy gap. Chi-square test showed no significant difference in gender and incidence of smoking, alcohol consumption, hypertension, diabetes, history of aspirin, thrombotic events, blood transfusion requirement, and wound hematoma and infection (Table 1).

**Figure 1:**
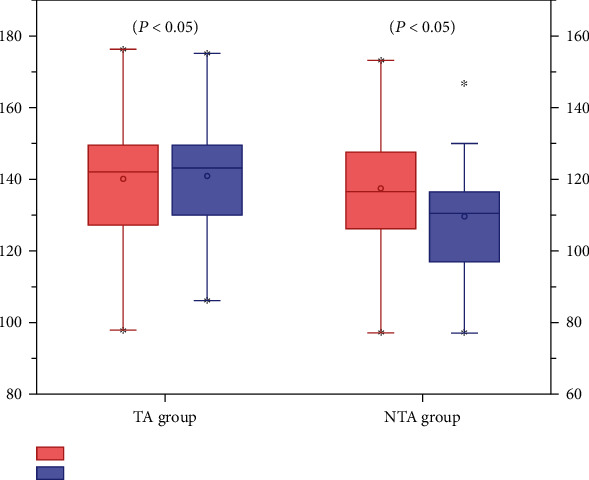
Hb levels decreased significantly in both groups.

Hb levels decreased significantly after OWHTO in both groups (Figure 1). The mean hospital length of stay was 9.4 ± 1.0 days in the TA group and 11.0 ± 1.2 days in the NTA group (*P* < 0.001), the blood loss was 296.0 ± 128.7 ml in the TA group and 383.3 ± 181.3 ml in the NTA group (*P* < 0.05), and the postoperative Hb level was 120.8 ± 15.0 g/l in the TA group and 109.5 ± 13.8 g/l in the NTA group (*P* < 0.001). There were significant differences in hospital length of stay, blood loss, and postoperative Hb in both groups (Table 2).

Multiple linear regression model suggested alcohol consumption and BMI were associated with blood loss in HTO (P < 0.05).

## 4. Discussion

OWHTO is an accepted and reliable procedure for the treatment of medial compartmental osteoarthritis in young, active individuals with varus deformity [8, 9, 11–13, 30]. However, during OWHTO surgery, extensive soft tissue destruction and osteotomy gap creating induce intraoperative and postoperative bleeding, which prolong recovery time and enhance the risk of infection [12–14]. In our previous study, we confirmed that the blood loss was 383.3 ± 181.3 ml in patients undergoing OWHTO [27].

In recent years, TXA has been widely used to reduce blood loss in knee surgery especially in total knee arthroplasty [31–34]. Meanwhile, there is limited research demonstrating that TXA was effective in reducing blood loss after OWHTO. Palanisamy et al. investigated 156 patients undergoing OWHTO and pointed out that the blood loss was 372 ± 36 ml in the TA group and 635 ± 53 ml in the NTA group [35]. Similarly, Kim et al. investigated 150 patients undergoing OWHTO and pointed out that the blood loss was 502.4 ± 294.9 ml in the TA group and 882.7 ± 482.0 ml in the NTA group [36].

Our findings suggest that Hb levels decreased significantly in both groups after OWHTO; The TA group exhibits significantly higher postoperative Hb levels and lower blood loss compared with the NTA group. Besides, our research has once again demonstrated that alcohol consumption and BMI are important risk factors related to blood loss. Alcohol can disturb the coagulation system at several levels, causing abnormally low platelet numbers such as thrombocytopenia, impaired platelet function such as thrombocytopathy, and diminished fibrinolysis [37–39]. Body surface area and soft tissue were larger in patients with higher BMI [40], which might cause more damage of the soft tissue in OWHTO. Meanwhile, during anaesthesia, increased ventilation pressures required to overcome lower pulmonary compliance in higher BMI patients may result in greater blood loss through higher venous pressures and exudation [41].

Another interesting result from this study is that the administration of TXA can reduce hospital length of stay; a reasonable explanation is that the administration of TXA can decrease joint swelling, promote physical activity, and secure earlier discharge [42–44]. In a study conducted by Floerkemeier et al., 533 patients undergoing OWHTO were reviewed; the hospital length of stay was 8.5 days [45]. Kriegshauser and Bryan pointed out that the hospital length of stay of patients was 10.7 days [46]. In our study, the mean hospital length of stay was 9.4 ± 1.0 days in the TA group and 11.0 ± 1.2 days in the NTA group.

In our study, we have got a remarkable result about the efficacy of TXA in patients undergoing OWHTO; however, the optimal dose and route of administration of TXA in OWHTO are still debated. Palanisamy et al. suggested patients received an intraoperative infusion of TXA 2 g before tourniquet application, and the same amount was repeated in 3 hours [35]. Differently, Suh et al. proposed that topical TXA 2 g can be administered at the osteotomy site [14]. Moreover, Kim et al. pointed out that 10 mg/kg of TXA can be administered via intravenous injection three times [36]. Peak plasma concentrations of TXA are obtained in 5–15 min for intravenous infusion, and the biological half-life is about 2 hours; correspondingly, the operation time of OWHTO is usually within 2 hours [47–49]. Topical TXA can provide a high local concentration, arrest bleeding directly, and reduce blood loss rapidly [48]. Based on the above analysis, the patients in our study received intravenous combined with topical administration of TXA. Overall, our study concluded that the administration of TXA is beneficial to patients undergoing OWHTO via decreasing hospital length of stay, reducing blood loss, and maintaining higher postoperative Hb levels.

This study has several limitations. First, it is a small retrospective study. Second, our research is a single-center study; the results may be biased, and a further larger, multicenter study was needed to confirm our findings.

## 5. Conclusions

Conclusions should clearly explain the main findings and implications of the work, highlighting its importance and relevance.

## Figures and Tables

**Table 1:** Patient demographics.

	TA group	NTA group.	*P* value
Gender (male/female)	20/32	22/26	0.46
Mean age (years)	58.3 ± 10.4	56.6 ± 10.2	0.43
BMI	27.3 ± 4.0	28.5 ± 4.2	0.18
Pre Hb (g/l)	140.0 ± 16.5	137.4 ± 15.0	0.40
Osteotomy gap (mm)	10.3 ± 3.6	10.9 ± 3.3	0.36
Smoking	14 (26.9%)	13 (27.1%)	0.99
Alcohol consumption	16 (30.8%)	15 (31.2%)	0.96
Hypertension	25 (48.1%)	23 (47.9%)	0.99
Diabetes mellitus	9 (17.3%)	10 (20.8%)	0.65
History of aspirin	12 (21.2%)	12 (25.0%)	0.65
Thrombotic events	2 (3.8%)	4 (8.3%)	0.35
Hematoma	2 (3.8%)	5 (10.4%)	0.20
Infection	1 (1.9%)	2 (2.1%)	0.95

**Table 2:** Significant difference between both groups.

	TA group	NTA group	*P* value
Pos Hb (g/l)	120.8 ± 15.0	109.5 ± 13.8	0.00
Blood loss (ml)	296.0 ± 128.7	383.3 ± 181.3	0.01
hospital length of stay (days)	9.4 ± 1.0	11.0 ± 1.2	0.00

## Data Availability

The datasets generated during and/or analyzed during the current study are available from the corresponding author on reasonable request.

## References

[1] Zhou M., Guo Y., Wang D. (2017). The cross-sectional and longitudinal effect of hyperlipidemia on knee osteoarthritis: results from the Dongfeng-Tongji cohort in China. *Scientific Reports*.

[2] Huang T.-C., Chang W.-T., Hu Y.-C. (2018). Zinc protects articular chondrocytes through changes in Nrf2-mediated antioxidants, cytokines and matrix metalloproteinases. *Nutrients*.

[3] Huber R., Kirsten H., Näkki A. (2019). Association of human FOS promoter variants with the occurrence of knee-osteoarthritis in a case control association study. *International Journal of Molecular Sciences*.

[4] Arjmand H., Nazemi M., Kontulainen S. A. (2019). Author correction: mechanical metrics of the proximal tibia are precise and differentiate osteoarthritic and normal knees: a finite element study. *Scientific Reports*.

[5] Cross M., Smith E., Hoy D. (2014). The global burden of hip and knee osteoarthritis: estimates from the global burden of disease 2010 study. *Annals of the Rheumatic Diseases*.

[6] Buckwalter J. A., Martin J. A. (2006). Osteoarthritis. *Advanced Drug Delivery Reviews*.

[7] GBD 2015 DALYs and HALE Collaborators (2016). Global, regional, and national disability-adjusted life-years (DALYs) for 315 diseases and injuries and healthy life expectancy (HALE), 1990-2015: a systematic analysis for the Global Burden of Disease Study 2015. *Lancet*.

[8] Kim J.-H., Kim H.-J., Lee D.-H. (2017). Survival of opening versus closing wedge high tibial osteotomy: a meta-analysis. *Scientific Reports*.

[9] W-Dahl A., Robertsson O., Lohmander L. S. (2012). High tibial osteotomy in Sweden, 1998-2007: a population-based study of the use and rate of revision to knee arthroplasty. *Acta Orthopaedica*.

[10] Astur D. C., Novaretti J. V., Gomes M. L. (2020). Medial opening wedge high tibial osteotomy decreases medial meniscal extrusion and improves clinical outcomes and return to activity. *Orthopaedic Journal of Sports Medicine*.

[11] Takeuchi R., Umemoto Y., Aratake M. (2010). A mid term comparison of open wedge high tibial osteotomy vs unicompartmental knee arthroplasty for medial compartment osteoarthritis of the knee. *Journal of Orthopaedic Surgery and Research*.

[12] O'Malley M., Reardon P. J., Pareek A., Krych A., Stuart M. J. (2016). Opening-wedge proximal tibial osteotomy. *Arthroscopy Techniques*.

[13] Pipino G., Indelli P. F., Tigani D., Maffei G., Vaccarisi D. (2017). Opening-wedge high tibial osteotomy: a seven - to twelve-year study. *Joints*.

[14] Suh D. W., Kyung B. S., Han S.-B., Cheong K., Lee W. H. (2017). Efficacy of tranexamic acid for hemostasis in patients undergoing high tibial osteotomy. *The Journal of Knee Surgery*.

[15] Ducloy-Bouthors A.-S., Jude B., Duhamel A. (2011). High-dose tranexamic acid reduces blood loss in postpartum haemorrhage. *Critical Care*.

[16] Novikova N., Hofmeyr G. J. (2010). Tranexamic acid for preventing postpartum haemorrhage. *Cochrane Database of Systematic Reviews*.

[17] Ker K., Beecher D., Roberts I. (2013). Topical application of tranexamic acid for the reduction of bleeding. *Cochrane Database of Systematic Reviews*.

[18] McCormack P. L. (2012). Tranexamic acid: a review of its use in the treatment of hyperfibrinolysis. *Drugs*.

[19] Thompson J. F., Royle G. T., Farrands P. A., Najmaldin A., Clifford P. C., Webster J. H. (1990). Varicose vein surgery using a pneumatic tourniquet: reduced blood loss and improved cosmesis. *Annals of the Royal College of Surgeons of England*.

[20] Tavakoli N., Mokhtare M., Agah S. (2017). Comparison of the efficacy of intravenous tranexamic acid with and without topical administration versus placebo in urgent endoscopy rate for acute gastrointestinal bleeding: a double-blind randomized controlled trial. *United European Gastroenterology Journal*.

[21] Sander M., Spies C. D., Martiny V., Rosenthal C., Wernecke K.-D., von Heymann C. (2010). Mortality associated with administration of high-dose tranexamic acid and aprotinin in primary open-heart procedures: a retrospective analysis. *Critical Care*.

[22] Franchini M., Mengoli C., Cruciani M. (2018). Safety and efficacy of tranexamic acid for prevention of obstetric haemorrhage: an updated systematic review and meta-analysis. *Blood Transfusion*.

[23] Longo M. A., Cavalheiro B. T., de Oliveira Filho G. R. (2018). Systematic review and meta-analyses of tranexamic acid use for bleeding reduction in prostate surgery. *Journal of Clinical Anesthesia*.

[24] Hodgson S., Larvin J. T., Dearman C. (2015). What dose of tranexamic acid is most effective and safe for adult patients undergoing cardiac surgery?. *Interactive Cardiovascular and Thoracic Surgery*.

[25] Guo J., Gao X., Ma Y. (2019). Different dose regimes and administration methods of tranexamic acid in cardiac surgery: a meta-analysis of randomized trials. *BMC Anesthesiology*.

[26] Poeran J., Rasul R., Suzuki S. (2014). Tranexamic acid use and postoperative outcomes in patients undergoing total hip or knee arthroplasty in the United States: retrospective analysis of effectiveness and safety. *BMJ*.

[27] Zhu J.-W., Chen D.-S., Wang T.-F., Xie Y. (2020). Patient characteristics related to blood loss in high tibial osteotomy in novel multiple linear regression analysis. *BioMed Research International*.

[28] Nadler S. B., Hidalgo J. H., Bloch T. (1962). Prediction of blood volume in normal human adults. *Surgery*.

[29] Gross J. B. (1983). Estimating allowable blood loss: corrected for dilution. *Anesthesiology*.

[30] Day M., Wolf B. R. (2019). Medial opening-wedge high tibial osteotomy for medial compartment arthrosis/overload. *Clinics in Sports Medicine*.

[31] Fillingham Y. A., Ramkumar D. B., Jevsevar D. S. (2018). The efficacy of tranexamic acid in total knee arthroplasty: a network meta-analysis. *The Journal of Arthroplasty*.

[32] Xie J., Hu Q., Huang Q., Ma J., Lei Y., Pei F. (2017). Comparison of intravenous versus topical tranexamic acid in primary total hip and knee arthroplasty: an updated meta-analysis. *Thrombosis Research*.

[33] Liu Q., Geng P., Shi L., Wang Q., Wang P. (2018). Tranexamic acid versus aminocaproic acid for blood management after total knee and total hip arthroplasty: a systematic review and meta-analysis. *International Journal of Surgery*.

[34] Peng Zhang M. M., Jifeng Li M. M., Xiao Wang M. M. (2017). Combined versus single application of tranexamic acid in total knee and hip arthroplasty: A meta-analysis of randomized controlled trials. *International Journal of Surgery*.

[35] Palanisamy J. V., Das S., Moon K. H., Kim D. H., Kim T. K. (2018). Intravenous tranexamic acid reduces postoperative blood loss after high tibial osteotomy. *Clinical Orthopaedics and Related Research*.

[36] Kim K.-I., Kim H. J., Kim G. B., Bae S. H. (2018). Tranexamic acid is effective for blood management in open-wedge high tibial osteotomy. *Orthopaedics & Traumatology, Surgery & Research*.

[37] Ballard H. S. (1997). The hematological complications of alcoholism. *Alcohol Health and Research World*.

[38] Smith S., Fair K., Goodman A., Watson J., Dodgion C., Schreiber M. (2019). Consumption of alcohol leads to platelet inhibition in men. *American Journal of Surgery*.

[39] Stote K. S., Tracy R. P., Taylor P. R., Baer D. J. (2016). The effect of moderate alcohol consumption on biomarkers of inflammation and hemostatic factors in postmenopausal women. *European Journal of Clinical Nutrition*.

[40] Tió M., Basora M., Rios J. (2018). Severe and morbid obesity and transfusional risk in total knee arthroplasty: an observational study. *The Knee*.

[41] Bowditch M. G., Villar R. N. (1999). Do obese patients bleed more? A prospective study of blood loss at total hip replacement. *Annals of the Royal College of Surgeons of England*.

[42] Ishida K., Tsumura N., Kitagawa A. (2011). Intra-articular injection of tranexamic acid reduces not only blood loss but also knee joint swelling after total knee arthroplasty. *International Orthopaedics*.

[43] Huang Z. Y., Huang Q., Zeng H. J. (2019). Tranexamic acid may benefit patients undergoing total hip/knee arthroplasty because of haemophilia. *BMC Musculoskeletal Disorders*.

[44] Huang Z., Ma J., Shen B., Pei F. (2014). Combination of intravenous and topical application of tranexamic acid in primary total knee arthroplasty: a prospective randomized controlled trial. *The Journal of Arthroplasty*.

[45] Floerkemeier S., Staubli A. E., Schroeter S., Goldhahn S., Lobenhoffer P. (2013). Outcome after high tibial open-wedge osteotomy: a retrospective evaluation of 533 patients. *Knee Surgery, Sports Traumatology, Arthroscopy*.

[46] Kriegshauser L. A., Bryan R. S. (1985). Early motion with cast-brace after modified Coventry high tibial osteotomy. *Clinical Orthopaedics and Related Research*.

[47] Pilbrant A., Schannong M., Vessman J. (1981). Pharmacokinetics and bioavailability of tranexamic acid. *European Journal of Clinical Pharmacology*.

[48] Georgiev G. P., Tanchev P. P., Zheleva Z., Kinov P. (2018). Comparison of topical and intravenous administration of tranexamic acid for blood loss control during total joint replacement: Review of literature. *Journal of Orthopaedic Translation*.

[49] Soni A., Saini R., Gulati A., Paul R., Bhatty S., Rajoli S. R. (2014). Comparison between intravenous and intra-articular regimens of tranexamic acid in reducing blood loss during total knee arthroplasty. *The Journal of Arthroplasty*.

